# Industrial Semi-Supervised Dynamic Soft-Sensor Modeling Approach Based on Deep Relevant Representation Learning

**DOI:** 10.3390/s21103430

**Published:** 2021-05-14

**Authors:** Jean Mário Moreira de Lima, Fábio Meneghetti Ugulino de Araújo

**Affiliations:** Computer Engineering and Automation Department, Federal University of Rio Grande do Norte, 3000 Senador Salgado Filho Avenue, Natal, RN 59078970, Brazil; jean@dca.ufrn.br

**Keywords:** soft sensors, deep learning, stacked autoencoders, mutual information, LSTM

## Abstract

Soft sensors based on deep learning have been growing in industrial process applications, inferring hard-to-measure but crucial quality-related variables. However, applications may present strong non-linearity, dynamicity, and a lack of labeled data. To deal with the above-cited problems, the extraction of relevant features is becoming a field of interest in soft-sensing. A novel deep representative learning soft-sensor modeling approach is proposed based on stacked autoencoder (SAE), mutual information (MI), and long-short term memory (LSTM). SAE is trained layer by layer with MI evaluation performed between extracted features and targeted output to evaluate the relevance of learned representation in each layer. This approach highlights relevant information and eliminates irrelevant information from the current layer. Thus, deep output-related representative features are retrieved. In the supervised fine-tuning stage, an LSTM is coupled to the tail of the SAE to address system inherent dynamic behavior. Also, a k-fold cross-validation ensemble strategy is applied to enhance the soft-sensor reliability. Two real-world industrial non-linear processes are employed to evaluate the proposed method performance. The obtained results show improved prediction performance in comparison to other traditional and state-of-art methods. Compared to the other methods, the proposed model can generate more than 38.6% and 39.4% improvement of RMSE for the two analyzed industrial cases.

## 1. Introduction

Several hardware sensors supply data for monitoring and control process optimization in industrial production processes [1]. However, traditional sensors cannot measure a category of key variables, such as concentrations, melt index, and octane number, in real-time. Laboratory analysis and online analyzers present measurement delays and high cost, then they do not fulfill increasing industrial requirements [2]. Data-driven models named soft sensors have been developed as a successful alternative to the above-mentioned issue [3]. Basically, soft-sensing uses secondary variables (i.e., easy-to-measure variables) to estimate primary variables (i.e., hard-to-measure variables) [4,5]. Countless soft sensors have been designed using traditional methods: principal component regression (PCR) [6,7], partial least square (PLS) [8,9], support vector machine (SVM) [10,11], gaussian process regression (GPR) [12,13], artificial neural network (ANN) [14,15], and so on.

As mentioned before, measuring quality-related variables requires large intervals, and it can be a high-cost procedure. In this particular case, the labeled data is minimal, while the unlabeled data samples are abundant and easily obtained. Traditional methods require labeled data for training only. However, building models with a limited amount of labeled data demonstrates unsatisfying performance [16]. In such a case, semi-supervised methods present a viable alternative using labeled and unlabeled samples for soft-sensing [17,18,19]. The extensive volume of unlabeled data stores latent information, and when it is extracted and applied adequately, it can reveal meaningful features about the process data. As a consequence, model reliability enhances, and prediction performance improves. Therefore, a soft-sensing key factor is the feature representation of process data.

In recent years, deep learning-based models have demonstrated solid representation proficiency and succeeded in many computer science fields with innovative results included image processing, natural language processing, speech recognition, computer vision, etc. [20,21,22]. Among the most extensively used deep networks architectures are stacked autoencoder (SAE) [23,24], deep belief network (DBN) [25,26], convolutional neural network (CNN) [26,27] and long short-term memory (LSTM) [28,29]. For deep learning structures such as SAE, the greedy layer-wise unsupervised pre-training and supervised fine-tuning are very significant. The SAE weights are computed by the unsupervised pre-training are applied for the fine-tuning supervised stage, which is a more effective strategy than random weight initialization [30]. For this reason, several industrial case-applied soft sensors have been proposed based on SAE [30,31,32,33,34,35,36,37]. The cited-above successful applications of SAE-based deep learning demonstrate a strong ability for feature extraction. The unsupervised layer-wise pre-training and supervised fine-tuning procedures allow deep structures to outperform the prediction performance of traditional techniques for soft-sensing.

Based on the hypothesis of static process and steady state, proposed industrial soft sensors are static models. However, the dynamicity of industrial processes, which is always present, cannot be overlooked. For example, chemical processes are highly dynamic with the current state related to previous stages. Thus, time-related features of time-series recorded data matters.

The recurrent neural networks (RNN), specifically the LSTM, are suitable for time-series processing since its structure uses past-state information to set the present state. Regarding soft-sensing, LSTM is appropriate to handle industrial process dynamics by considering the previous condition to compute the current state and preserving high capability for assimilating inherent non-linearity of the process [38,39]. Recently, industrial scenarios are the study-case for soft-sensing based on LSTM. In [40,41], authors proposed soft sensors based on LSTM for quality prediction in wastewater treatment plants. Moreover, LSTM-based soft sensors to estimate key quality variables in the fermentation process and debutanizer column [42]. Also, proposed LSTM soft-sensor models for predicting boiling points of heavy naphtha and aviation kerosene in [43]. However, those works do not use unlabeled samples for unsupervised pre-training, which may cause poor feature representation. In [44,45], the researchers proposed structures that merged unsupervised hidden features mining and supervised dynamic modeling. A denoising autoencoder (DAE) extracts meaningful features that serve as inputs for an LSTM soft-sensor applied to Fluid Catalytic Cracking (FCC) unit [44]. Also, in [45], an Xgboost is used to select features, acting as an encoder to feed a soft-sensor based on LSTM that extracts dynamic information hidden in-process data. However, for soft-sensing, the extracted representations must be related to the target-output variables. Otherwise, the overall soft-sensing prediction performance does not enhance.

In this work, a novel semi-supervised soft-sensing approach based on deep relevant representation learning is proposed to cope with all the above-cited problems. Mutual information (MI), SAE, and LSTM integrate the proposed method named MISAEL. In the unsupervised pre-training phase, SAE is trained layer-by-layer using all available unlabeled and labeled data. After each layer training, MI analysis evaluates the learned representations by calculating a correlation coefficient between target-output and the current-layer output. This strategy eliminates irrelevant information, and the current layer retains representative information only. The process repeats itself until the last SAE layer, modeling an SAE structure with target-output-related representations only. Thus, the first stage of the proposal exploits the unlabeled data by extracting deeply hidden features, and then MI highlights the most relevant learned representations for soft-sensing purposes. Representative features are one of the main factors for industrial soft-sensor modeling. However, the SAE may not represent the inherent spatial-temporal dynamicity of the industrial process adequately. To accomplish such a task is necessary to model the time dynamic behavior for the final prediction. In the supervised fine-tuning phase, an LSTM couples to the last SAE layer. The entire deep architecture composed of pretrained MI-based SAE and the LSTM is trained using the labeled data. The proposed method allows MISAEL to extract hidden features and select the most relevant representations adaptively, also handle dynamic behavior properly. Therefore, the integrated in-depth learning-based approach can improve industrial soft-sensor prediction performance, refine robustness, and enhance reliability. The main contributions of this research are as follow:

1. A novel semi-supervised soft-sensor modeling based on deep representative learning is proposed to enhance soft-sensing prediction performance. The proposed method can be applied to soft sensors under scarce labeled data, high non-linearity, and dynamic behavior.
2. A deep representative learning method extracts high-level features from unlabeled data and then eliminates non-relevant representations and highlights relevant information for efficient soft-sensing development.
3. MI analysis evaluates the relation among targeted-output variables and an SAE model representations in a layer-by-layer manner. Thus, the pretrained deep architecture is more suitable and reliable for soft-sensing.
4. An LSTM model couples to the pretrained SAE to address the inherent dynamic features of the process. A soft-sensor specifically trained to handle systems dynamic outperforms other traditional and enhanced-SAE-based methods.

The above-mentioned contributions have been demonstrated acceptable and successful for soft-sensing by using two industrial plant study cases, a debutanizer column, and a sulfur recovery unit process. The rest of this paper is arranged as follows. In Section 2, preliminaries are described. Section 3 gives the details of the proposed approach, integrating the SAE, MI evaluation, and LSTM modeling. Industrial process case studies are used to evaluate the proposed method performance, and the results are present in Section 4. Finally, Section 5 summarizes the conclusions of the work.

## 2. Preliminares

### 2.1. Stacked Autoencoders

**Figure 1:**
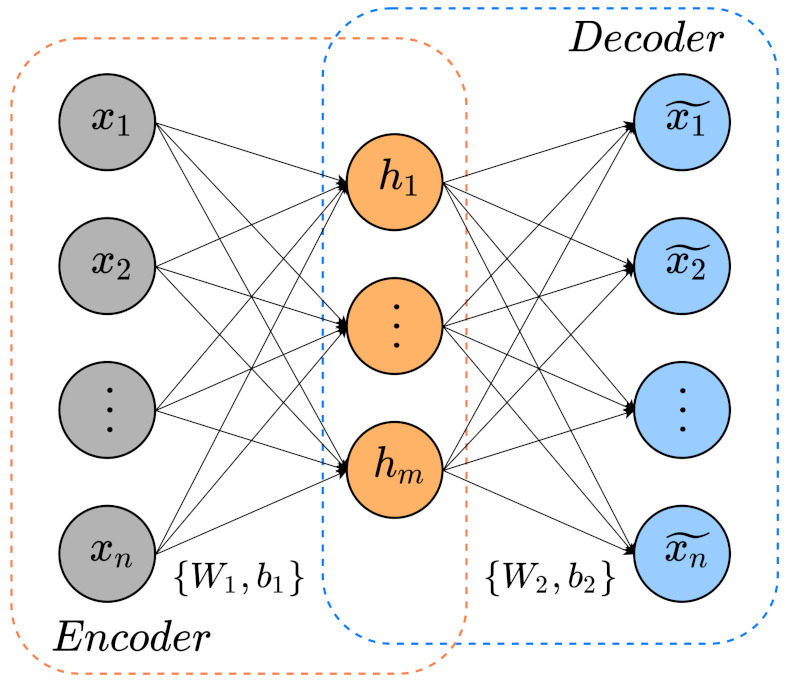
Basic AE schematic.

The autoencoders (AE) represent a network formed by three layers: an input layer, hidden layer, and output layer as Figure 1 describes. Although the encoder identifies low-dimensional features from the input data, the decoder exploits the extracted hidden features to rebuild the input data. An AE learns valuable features from data restoring input data as similar to the original input.

The encoder computes input $x={\left[x_{1},x_{2},\cdots ,x_{n}\right]}^{T}\in R^{n}$ and maps it into a low-dimensional hidden features $h={\left[h_{1},h_{2},\cdots ,h_{m}\right]}^{T}\in R^{m}$. In addition, the decoder processes the obtained hidden features to approximate the input data. Equations (1) and (2) describes the cited operations:

(1) $$h=f(W_{e}x+b_{e}),$$

(2) $$\hat{x}=g\left(W_{d}h+b_{d}\right),$$

where $W_{e}\in R^{n\times m}$, $b_{e}\in R^{m}$, $W_{d}\in R^{m\times n}$ and $b_{e}\in R^{n}$ are weight matrices and bias of the encoder and decoder, respectively. Terms *f* and *g* are the commonly used activation functions sigmoide or ReLU [46].

Normally, mean square error (MSE) between x and $\hat{x}$ is the loss function in the training of AE as Equation (3) demonstrates. The parameters set $\left(W_{e},b_{e},W_{d},b_{d}\right)$ is used to minimize the reconstruction error.

(3) $$J_{AE}=\frac{1}{m}\sum_{i=1}^{m}\left(\frac{1}{2}{∥\hat{x_{i}}-x_{i}∥}^{2}\right)$$

Furthermore, this work applies two strategies to enhance the overall AE performance. A weight of decay to avoid overfitting [34], and sparse restriction regularization to penalize hidden units with high activating ratio [47]. Equations (4) and (5) show both regularization techniques. In Equation (4), λ is the component to regulate $J_{WD}$ and $u_{l}$ indicates layer *l* units. Also, in Equation (5), β is the adjustment parameter, *m* is hidden layer units, ρ is the wanted activatin ratio, and $\rho_{j}$ is the average activation value for the *j*-th hidden layer neuron.

(4) $$J_{WD}=\frac{\lambda }{2}\sum_{l=1}^{2}\sum_{i=1}^{u_{l}}\sum_{j=1}^{u_{l+1}}{\left(w_{ji}\right)}^{2},$$

(5) $$J_{SR}=\beta \sum_{j}^{m}\rho \log\frac{\rho }{\rho_{j}}+\left(1-\rho \right)\log\frac{1-\rho }{1-\rho_{j}}.$$

The reconstruction loss function including the regularization parameters $J_{WD}$ and $J_{SR}$ are given as follows:

(6) $$J_{AE}=J_{AE}+J_{WD}+J_{SR}$$

**Figure 2:**
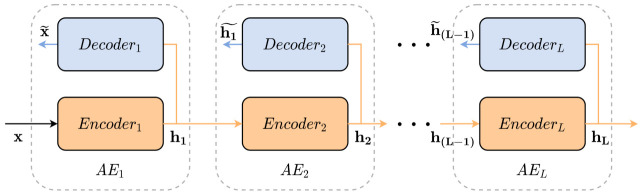
Stacked Autoencoders schematic diagram.

A deep structure able to learn high-level features is conceived by stacking several AE, and thus each AE is an SAE layer. As shown in Figure 2, the stacked autoencoder (SAE) uses the previous layer output to feed the next layer input. Two stages compose the SAE training, unsupervised pre-training and supervised fine-tuning. In the unsupervised phase, the pre-training layer-by-layer minimizes the reconstruction loss function Equation (6). In contrast, the supervised fine-tuning optimize all SAE parameters through prediction error minimization [48,49].

SAE uses unlabeled and labeled samples to implement semi-supervised soft sensors, but not necessarily the network learned meaningful representations for soft-sensing quality variables. Traditionally, unsupervised pre-training disregards target-output data, which may lead to irrelevant learned features for the prediction task [50]. The unimportant information distributed all over the layers can degrade prediction performance even after a successfully fine-tuning. Hence, the elimination of irrelevant representations in the pre-training stage can improve soft-sensing efficiency.

### 2.2. Mutual Information

Mutual Information (MI) evaluates the correlation between two random variables regarding entropy quantitatively [51,52]. MI describes the linear, periodic, or non-linear relationship among arbitrary variables, and thus it is more comprehensive than traditional methods such as correlation coefficient [53].

The MI between two given random variables X and Y is defined as follows [51]:

(7) $$MI\left(x,y\right)={\;\int \int \;}_{x,y}p\left(x,y\right)\log\left(\frac{p(x,y)}{p(x)p(y)}\right)dxdy,$$

where p(x) and p(y) represent the marginal probability distributions, and p(x,y) is the joint probability distribution between *x* and *y*. Moreover, Shannon entropy of a random variable *x* is described as H(x) [51]:

(8) $$H\left(x\right)=-\int_{x}p\left(x\right)\log p\left(x\right)dx.$$

Then, Equation (7) becomes the following:

(9) MI(x,y)=H(x)+H(y)−H(x,y),

where H(x,y) corresponds to the joint entropy between the variables *x* and *y*. H(x,y) is computed as follows [51]:

(10) $$H\left(x,y\right)=-{\;\int \int \;}_{x,y}p\left(x,y\right)\log\left(p\left(x,y\right)\right)dxdy.$$

According to the equations above, estimating the probability density functions (PDFs) is necessary to compute MI values. Both parametric and non-parametric procedures can calculate the PDFs, but the PDF estimation is not a simple task in practical applications [54]. K-nearest neighbor (K-NN) non-parametric method to calculate MI was proposed in [51]. Through the above-cited technique, calculation complexity of MI decreases since it relies upon the given data only. Hence, this work adopts the (K-NN) method for MI calculation.

### 2.3. Long-Short Term Memory

Long-Short Term Memory (LSTM) is an improved version of RNNs which replaces hidden neurons for LSTM units. LSTM unit is named memory cell $c_{t}$, which is the core of LSTM and retains previous time-step information. Three gate structures compose the LSTM unit, namely input gate, forget gate, and output gate. The gates capture both short-term and long-term memory and control the portion of information to keep or relieve in the subsequent time step. Beyond preserving RNN advantages, LSTM proves enhanced performance when dealing with time-series. Thus, LSTM has modeled time-series applications to handle non-linear dynamics lately.

**Figure 3:**
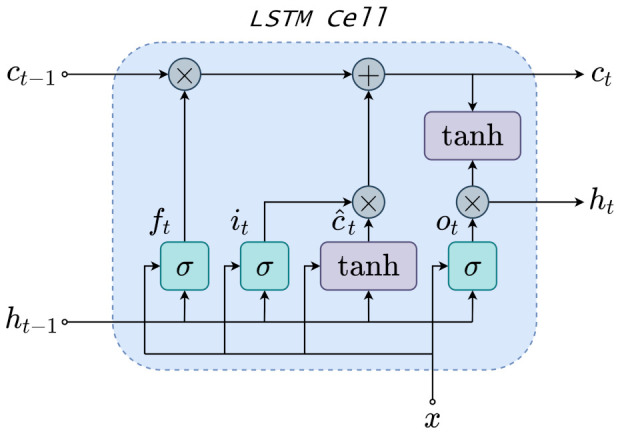
LSTM Cell schematic.

The Figure 3 illustrates a detailed LSTM cell, which present three gate controllers namely the input $i_{t}$, forget $f_{t}$, and output $o_{t}$ gates. The three gates decide the information that should be remembered or not, and σ represent their activation function. For the basic LSTM unit in Figure 3, external inputs are the input vector *x*, previous hidden state $h_{t-1}$, and previous cell state $c_{t-1}$. An intermediate state ${\hat{c}}_{t}$ is generated inside the LSTM, which is part of computation of the current cell state $c_{t}$. The LSTM model aims to obtain a latent variable $h_{t}$ to provide valuable dynamic information. To cope with this issue, LSTM gates are developed. The input gate $i_{t}$ of the LSTM cell is given as follows:

(11) $$i_{t}=\sigma \left(W_{xi}x_{t}+W_{hi}h_{t-1}+b_{i}\right),$$

where σ is the sigmoid activation function, $x_{t}$ is the input vector and $h_{t-1}$ represents previous state latent variables. The $W_{xi}$, $W_{hi}$ are the weighting matrices for $x_{t}$ and $h_{t-1}$ in the input gate, respectively. Finally, $b_{i}$ is the bias. In addition, tanh activation function defines an intermediate state ${\tilde{c}}_{t}$ to gather important fraction of the input as follows:

(12) $${\tilde{c}}_{t}=\tanh\left(W_{xc}x_{t}+W_{hc}h_{t-1}+b_{c}\right),$$

where $W_{xc}$, $W_{hc}$ are the weighting matrices and $b_{c}$ is the bias. Furthermore, the forget gate defines whether the long-term memory from previous cell remains or not as follows:

(13) $$f_{t}=\sigma \left(W_{xf}x_{t}+W_{hf}h_{t-1}+b_{f}\right),$$

where $W_{xf}$, $W_{hf}$ are the weighting matrices and $b_{f}$ represents the bias. Therefore, the cell state $c_{t}$ aggregates long-term memory retained information and weighted input. The $c_{t}$ is defined as:

(14) $$c_{t}=f_{t}⊙c_{t-1}+i_{t}⊙{\tilde{c}}_{t},$$

where $c_{t-1}$ is the previous cell state, and ⊙ denotes pointwise multiplication. Moreover, the output gate connects hidden latent state and cell state to establish a relation between them. The output gate is denoted as:

(15) $${\tilde{o}}_{t}=\tanh\left(W_{xo}x_{t}+W_{ho}h_{t-1}+b_{o}\right),$$

where $W_{xo}$, $W_{ho}$ are the weighting matrices and $b_{o}$ points the bias. The pointwise multiplication between $c_{t}$ and $o_{t}$ formulates the current hidden latent state $h_{t}$ as follows:

(16) $$h_{t}=o_{t}⊙\tanh\left(c_{t}\right).$$

Finally, the estimated output ${\hat{y}}_{t}$ is given on the basis of hidden latent state as:

(17) $${\hat{y}}_{t}=\sigma \left(W_{y}h_{t}+b_{y}\right),$$

where $W_{y}$ represents the weighting matrix and $b_{y}$ denotes the output bias.

In summary, the Equations (11)–(17) demonstrate the LSTM forward pass network. LSTM can describe temporal dynamic behavior, which turns LSTM suitable for non-linear dynamic soft-sensing modeling. Moreover, LSTM better handle the vanishing gradient problem over the backpropagation through time (BPTT) iterations. Further details about BPTT are found in [55].

## 3. The Proposed MISAEL Method

This section details the concept and design of the MISAEL-based soft-sensor step-by-step. The unsupervised pre-training phase implements an MI-based layer-by-layer SAE, which learns relevant information and eliminates irrelevant representations. In the supervised fine-tuning, an LSTM structure couples to the MI-based SAE. The LSTM can learn the non-linear dynamic behavior of the process regarding its potential in dealing with time-series problems.

### 3.1. Data Preprocessing

Data preprocessing normalizes all collected data {X,Y} to a [0,1] range, which gives more stability to the model. The dataset is split into two groups: unlabeled $\left\{X^{U}\right\}$ and labeled $\left\{X^{L},Y\right\}$ data with ratios of 90% and 10%, respectively. This ratio difference aims to emulate process situations where labeled data is scarce but unlabeled abundant. The unsupervised pre-training uses only unlabeled data to reconstruct input and then induces the model to extract rich hidden features from this non-labeled data. Also, the pre-training stage uses 10% of unlabeled data for model validation.

The labeled data $\left\{X^{L},Y\right\}$ is distributed into 3 subsets: ${\left\{X^{L},Y\right\}}_{Tr}$ training set, ${\left\{X^{L},Y\right\}}_{V}$ validation set, and ${\left\{X^{L},Y\right\}}_{Te}$ testing set, with ratios of 40%, 10%, and 50%, respectively. Supervised fine-tuning uses the three labeled subsets to train and test the entire deep architecture MISAEL.

### 3.2. Unsupervised Pre-training: MI-Based SAE

The reconstruction of input data at the output layer is the main objective of a common AE. Through the minimization of the loss function in Equation (6), AE learns representations layer-by-layer. To reconstruct the inputs, all samples in the dataset are equally considerable for regular applications. For soft-sensing, instead, not all variables are similarly relevant to construct AEs with meaningful representations. Regarding the inference of target-output values, non-relevant information can disturb predictions since they are present in each AE layer and interfere in the final output.

This work employs MI to evaluate representations to cope with the above-cited issue. The Equation (7) calculates the MI among each variable and targeted outputs. When the calculated MI is smaller than a threshold value, the processed variable $x_{i}$ is not relevant as follows:

(18) $$MI(x_{i},y)\leq th,$$

where th is the threshold value. The th is the minimum relevance required to pass a variable or remove it when its MI is less than th. A MI value between an arbitrary signal, which is unrelated to the targeted output, and the target-output variables determine the th.

The MISAEL unsupervised pre-training can be divided into steps as follows:

Step 1. The first step is the calculation of an effectual MI threshold value. A 1000 random vectors are generated under an uniform distribution with values range of [0,1]. MI analysis between the generated arbitrary vectors and the targeted output is performed. MI values are sorted in descending order, and the 50th value indicates the threshold th value. Therefore, MI analysis obtains a confidence level of 95% when the MI value is higher than th. In the proposed method, th is not adaptive, and its value does not change during the entire training process.
Step 2. By using the labeled training dataset ${\left\{X^{L},Y\right\}}_{Tr}$, MI analysis indicates the relevance of all process variables. The procedure eliminates irrelevant variables to use only preserved variables in the training of the first AE. Retained unlabeled variables $x^{R}=\left\{X_{r}^{U}\right\}$ are used to train the first AE as follows:
  (19) $$h_{1}=f\left(W_{1}x^{R}+b_{1}\right)$$
  where $h_{1}$ is the hidden representation of the first AE. According to Equation (6) the first AE is trained.
Step 3. As hidden representations of first AE $h_{1}$ are computed, MI evaluation is performed to remove irrelevant representations and retain important information only. By using ${\left\{X^{L}\right\}}_{Tr}$, hidden representations $h_{1}^{L}$ are calculated to perform MI analysis. The MI between each $h_{1}^{L}$ representation and the corresponding target-output ${\left\{Y\right\}}_{Tr}$ is evaluated: $MI\left(h_{1}^{L},Y\right)\leq th$. According to MI values, the procedure wipes out the respective lines of data in the weighting matrix $W_{1}$ and bias $b_{1}$ that corresponds to non-relevant hidden representations, generating a new parameters set $\left\{W_{1}^{R},b_{1}^{R}\right\}$. Then unimportant representations are eliminated while meaningful representations are kept and used as input of the second AE.
Step 4. By reiterating the procedure in the previous step over the *L* stacked AE, high-level representative information is obtained over all the deep structure.
  (20) $$h_{L}=f\left(W_{L}h_{\left(L-1\right)}^{R}+b_{L}\right)$$
  where $h_{L}$ is the hidden representation of the *L*-th AE, $W_{L}$, $b_{L}$ are parameters set of AE-L, and $h_{L-1}^{R}$ the retained representations of the AE-(L-1). A set of optimized parameters $\left\{W_{1}^{R},b_{1}^{R},\cdots ,W_{L}^{R},b_{L}^{R}\right\}$ is acquired. Therefore, this procedure implements an MI-based SAE with soft-sensing relevant representations only.

**Figure 4:**
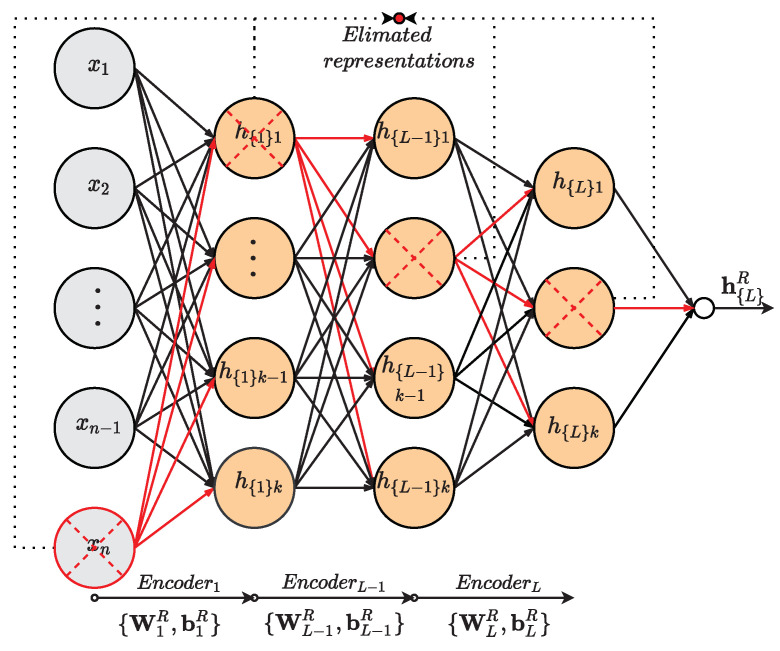
MI-base relevant representation approach.

The presented model removes non-relevant information present in the parameters set, and as a result, it emphasizes pertinent representations regarding soft-sensor operation. The Figure 4 illustrates the described process. Hence, the model can predict outputs with better performance and improved reliability.

### 3.3. Supervised Fine-Tuning: MI-SAE-LSTM

The *L*-th layer of the designed MI-based SAE corresponds to high-level relevant extracted features after the pre-training stage. However, the model may not learn the inherent dynamic behavior of the system even after the supervised fine-tuning phase. An LSTM is coupled to an MI-based SAE structure to accomplish the supervised fine-tuning and obtain a soft-sensor capable of dealing with real application dynamicity. The LSTM inputs are the meaningful features present in MI-SAE top layer. Moreover, before the fine-tuning phase begins, a k-fold cross-validation strategy is applied.

The supervised fine-tuning stage is split into steps as follows:

Step 1. The relevant parameters set $\left\{W_{1}^{R},b_{1}^{R},\cdots ,W_{L}^{R},b_{L}^{R}\right\}$ obtained in the unsupervised pre-training initializes the SAE model for the supervised fine-tuning. As a result, at layer *L*, high-level relevant extracted features Φ constitute the output the SAE and then the input of the next coupled structure.
Step 2. An LSTM model is coupled to the *L*-th layer of the SAE to address the process dynamics. Thus, supervised fine-tuning is performed in the entire established deep architecture. As the SAE feeds the LSTM, the Equations (11)–(13) and (15) are updated as follows:
  (21) $$i_{t}=\sigma \left(W_{xi}\Phi +W_{hi}h_{t-1}+b_{i}\right),$$
  (22) $${\tilde{c}}_{t}=\tanh\left(W_{xc}\Phi +W_{hc}h_{t-1}+b_{c}\right),$$
  (23) $$f_{t}=\sigma \left(W_{xf}\Phi +W_{hf}h_{t-1}+b_{f}\right),$$
  (24) $${\tilde{o}}_{t}=\tanh\left(W_{xo}\Phi +W_{ho}h_{t-1}+b_{o}\right).$$
Step 3. The k-fold cross-validation uses the training set ${\left\{X^{L},Y\right\}}_{Tr}$ to generate *k* subsets randomly. One of the *k* subsets composes the validation set, and the remaining *k*−1 subsets train the deep model. This procedure is then repeated *k* times with each of the *k* subsets used exactly once as the validation set.
Step 4. The previous step generated *k* MISAEL candidate deep models to compose the soft-sensor. The output of each candidate model ${\hat{y}}_{i}$ is correspondingly weighted to compute ensemble prediction of MISAEL $\hat{y}$.
  (25) $$\hat{y}=\frac{1}{k}\sum_{i=1}^{k}{\hat{y}}_{i},$$
  where *k* is the number of generated MISAEL candidates.

**Figure 5:**
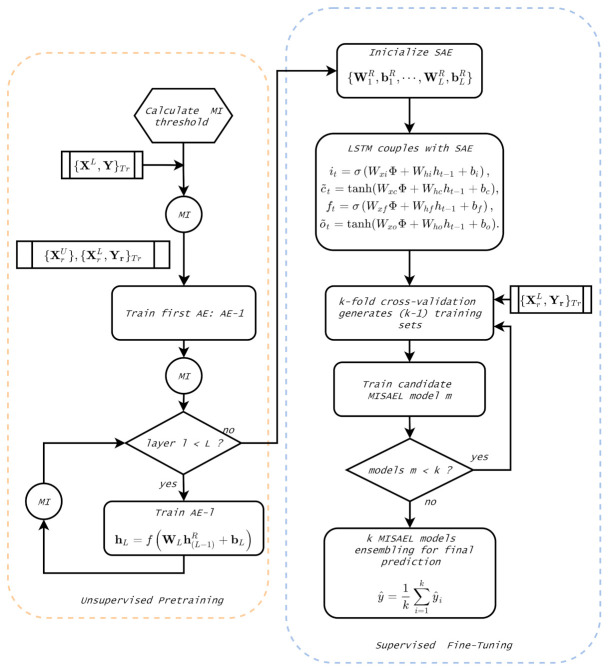
Proposed MISAEL method flowchart.

Figure 5 illustrates the summarized procedure to build proposed method MISAEL. In the unsupervised pre-training phase, an MI-SAE trained layer-by-layer until the *L*-th layer. MI analysis eliminates irrelevant information and, consequently, keeps relevant representation for each layer of the SAE. Moreover, an LSTM network couples to the tail of the acquired MI-SAE. The supervised fine-tuning of the entire deep architecture is performed by using a k-fold cross-validation strategy. Thus, *k* candidate MISAEL models are created, and then their output is aggregated to constitute the output.

## 4. Case Studies and Results

Through a debutanizer column and a sulfur recovery unit (SRU) processes the MISAEL performance is tested. Models used for comparison purposes are as follows:

1. traditional learning methods: PLS, MLP, and SVR.
2. Deep learning-based methods: SAE.
3. Proposed deep relevant learning-based soft-sensor: MISAEL and eMISAEL (ensemble MISAEL) designed by using the proposed soft-sensing method.

The root-mean-square error (RMSE) and coefficient of determination ($R^{2}$) are the applied metrics to quantify the prediction efficiency of the developed soft-sensing methods:

(26) $$RMSE=\sqrt{\frac{1}{N_{Ts}}\sum_{i=1}^{N_{Ts}}{\left({\hat{y}}_{i}-y_{i}\right)}^{2}}$$

(27) $$R^{2}=1-\frac{\sum_{i=1}^{N_{Ts}}{\left({\hat{y}}_{i}-y_{i}\right)}^{2}}{\sum_{i=1}^{N_{Ts}}{\left(y_{i}-{\bar{y}}_{i}\right)}^{2}}$$

where $y_{i}$ and ${\hat{y}}_{i}$ are the real and predicted outputs, respectively. The $\bar{y}$ represents the mean value, and $N_{Ts}$ denotes the number of samples in the testing set.

RMSE evaluates the prediction error and is traditionally used to assess the prediction performance of soft-sensing methods. This metric measures the overall expected deviation between predicted and actual values in a squared error sense [56]. Therefore, RMSE highly reflects the prediction performance and reliability of soft sensors to be tested [12]. A small RMSE score indicates better generalization and prediction performance. Also, regarding the inherent uncertainty of predicting quality-related process variables, the standard deviation of the attained results over different runs is adopted as the uncertainty range metric. This approach is widely accepted and has been adopted as one of the evaluation metrics of sensors and soft sensors [57,58]. The $R^{2}$ represents the correlation among predicted and actual outputs [59]. $R^{2}$ value provides the total variance that can be clarified about the targeted-output by a model. As a result, a high $R^{2}$ indicates better performance, and the reliability of the model can be reflected by this index as well [30].

### 4.1. Industrial Debutanizer Column Process

**Figure 6:**
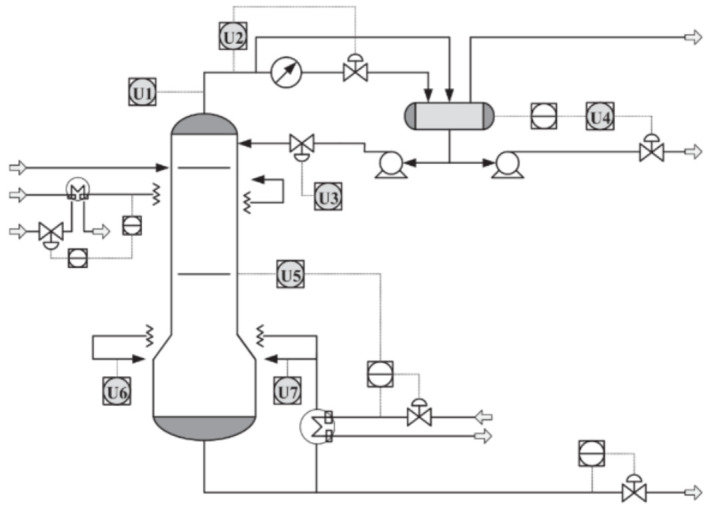
Schematic representation of the debutanizer column process [3].

Debutanizer columns are applied for desulfurization and naphtha cracking in an industrial refinery. A debutanizer column attempts to withdraw propane (C5) and butane (C4) from the naphtha stream [60,61]. Process performance improves when butane content is reduced, and then high-quality naphtha final products are acquired. However, hardware sensors are not able to measure butane content in real-time. As an option, soft-sensing is an interesting approach to infer butane concentration online. Figure 6 illustrates the primary flowchart of the debutanizer column used in this work. The main devices are the heat exchanger, head reflux pump, bottom reboiler, overhead condenser, head reflux pump, feed pump to the splitter, and reflux accumulator. In Figure 6, gray circles indicate the several hardware sensors that measure the process quality variables on the plant. The described debutanizer column aims to eliminate C3 and C4 from the naphtha stream. By minimizing the C4 concentration in the bottom of the debutanizer, product quality improves. However, gas chromatographs measure C4 concentration, and they do not provide online C4 concentrations for real-time control due to interval delays. As an alternative, soft sensors can handle difficulties in real-time measurements, providing real-time estimations of C4 concentration for real-time process control. In [3], process dataset is available as well as further details.

In real industrial scenarios as chemical process, labeled data is normally limited. From the debutanizer column, a total of 2384 samples were collected, but only 10% of samples denote the labeled set. The 90% of samples left represent the unlabeled dataset. The unsupervised pre-training uses the unlabeled samples as follows: 80% for training, 20% for validation. Moreover, three parts divide the labeled dataset: 100 samples for supervised fine-tuning, 20 samples for parameter optimization, and 120 samples for testing. PLS, MLP, SVR, and SAE soft-sensor models use all process variables from the labeled set as the input. MISAEL and eMISAEL models use selected inputs by performing MI analysis. Debutanizer column process variables are defined in Table 1. Furthermore, feature engineering is employed to add features that may incorporate former feature values to deal with process dynamics. Hence, soft-sensor inputs can be described as $X=[u\left(t\right),\cdots ,u\left(t-d_{x}\right),y\left(t-1\right),\cdots ,y\left(t-d_{y}\right)]$, where $d_{x}$ and $d_{y}$ represents the maximal delayed interval. In this work, $d_{x}=d_{y}=6$.

Through the strategy described in Section 3, the MI threshold is calculated and set to 0.076. SAE includes three AEs with 52, 49, and 46 hidden neurons at the beginning of the unsupervised pre-training stage. Hyperparameters as activation function, learning rate, and batch size are set as ReLU, 0.00085, and 30, respectively. The LSTM structure with 128 cell units is added to the SAE for fine-tuning and dynamic features learning after unsupervised pre-training. Also, three strategies to avoid overfitting are applied: early stopping, L2 regularization, and cross-validation.

**Figure 7:**
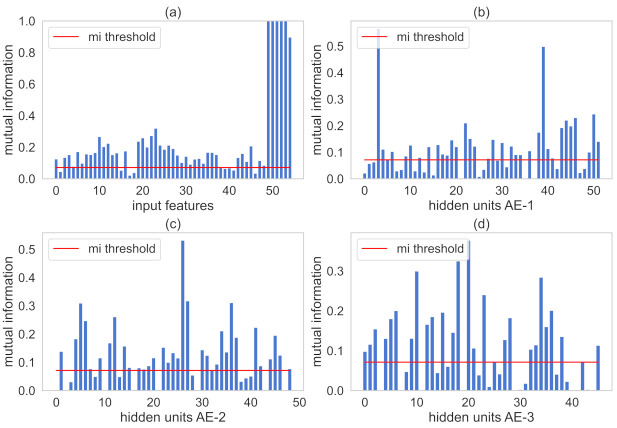
MI between input representations and the output variables for the debutanizer column. Subfigure (**a**) illustrates MI values of the input features. In addition, subfigures (**b**–**d**) indicate MI values hidden units present in AE-1, AE-2, AE-3, respectively.

Figure 7 illustrates the calculated MI values for the representation of the AE where the red line is the th value. MI values between input and the output variables are plotted in Figure 7a. The MI values among hidden representations of each AE and the output are plotted in Figure 7b–d, respectively. Variables are retained in the respective AE structure when its calculated MI values are greater than th.

Table 2 describes and compares the prediction performance among traditional models, state-of-art SAE-based models [33,62], and the proposed model MISAEL. The linear method PLS leads to the worst performance compared to all the other techniques that handle non-linearities. By using the unlabeled dataset in the pre-training, SAE-based models perform better than MLP and SVR, which do not use unlabeled data. As intended, MISAEL presents enhanced prediction performance compared to the SAE structure. MISAEL only has relevant representations within its acquired knowledge, turning MISAEL more suitable for soft-sensing. Moreover, the performance prediction of two state-of-art SAE-based methods was tested using the same debutanizer column process used in this work. These two methods are Hybrid VW-SAE [33], and SSED [62], and they are compared to MISAEL to strengthen the proposed method. According to the quantitative comparison illustrated in Table 2, MISAEL outperforms both HVW-SAE and SSED. HVW-SAE and SSED are enhanced-SAE structures as MISAEL, but MISAEL uses an LSTM model to handle the inherent dynamic behavior, which explains its improved performance. In addition, eMISAEL and MISAEL present the lowest standard deviations (SD) of RMSE, which indicates their stability under uncertain conditions. Finally, MISAEL and eMISAEL point to the best results in comparison to other traditional and SAE compared soft sensors methods. Also, MISAL outperforms two state-of-art methods [33,62] under same test conditions. The use of unlabeled data for pre-training, MI-SAE with relevant representations only, and coupled LSTM for fine-tuning are the three predominant benefits exploited for the MISAL model. Furthermore, eMISAEL exploits one more advantage, a k-fold cross-validation ensemble strategy which improves MISAEL prediction performance even more.

**Figure 8:**
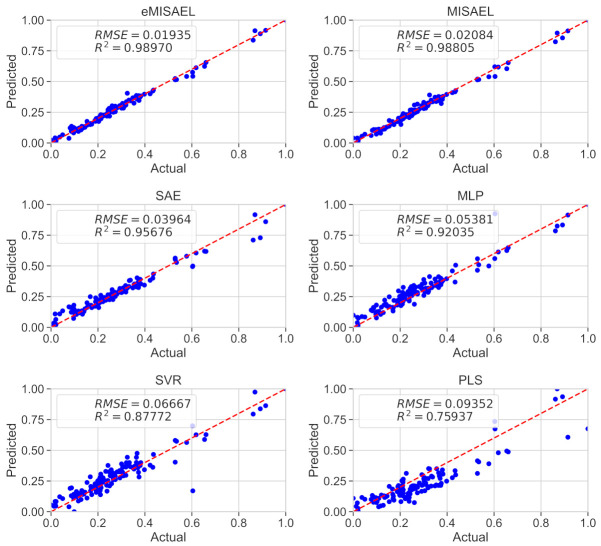
Real values of butane content and the predicted values using eMISAEL, MISAEL, SAE, MLP, SVR, and PLS soft-sensor models.

**Figure 9:**
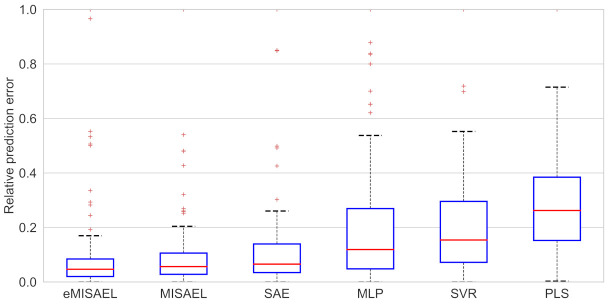
Relative prediction error of testing results for debutanizer column process using eMISAEL, MISAEL, SAE, MLP, SVR, and PLS models, respectively.

In Figure 8, the parity plots illustrate the achieved prediction results using the testing dataset. As expected, eMISAEL and MISAEL show more accuracy than the other methods. Furthermore, Figure 9 illustrates the relative prediction errors with boxplots of the six techniques eMISAL, MISAEL, SAE, MLP, SVR, and PLS in descending performance order, respectively. Inside each box, the central red mark represents the median value, and the edges of the box indicate the 25th and 75th percentiles. Above and below the box, the “whiskers” represent maximum and minimum values that are not outliers. The wider is the width of the box, the more dispersed prediction errors are. The narrowest box ranges of eMISAEL and MISAEL indicate the best prediction performance between the six compared methods mainly because MISAEL extracts non-linear features, selects the most relevant representations, and copes with the dynamicity of the process. Figure 9 plots a few outliers individually represented by red points as industrial processes contain uncertainties that should be considered [63]. High relative prediction errors may result from inadequate soft sensors due to both unsuitable initial parameters and outliers. Instead, an acceptable soft-sensor gives stationary relative prediction errors on the same data. Therefore, stationery predictions reflect the robustness of the model as well [64]. As a result, MISAEL and eMISAEL implement a soft-sensor with improved performance, enhanced reliability, and stronger robustness.

### 4.2. Sulfur Recovery Unit Process

**Figure 10:**
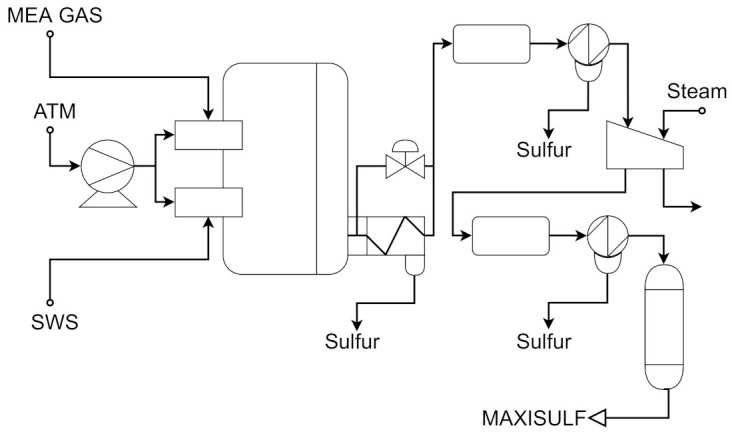
Simplified scheme of a SRU line based on [65].

In an industrial oil refinery, an acceptable sulfur emission rate is vital and, to accomplish such a task, a sulfur recovery unit (SRU) is employed. The SRU removes environmental pollutants and deals with the acid gas streams adequately. In this work, the chosen SRU unit is used in [65,66]. This SRU consists of four sulfur-lines subunits that transform acid gasses MEA and SWS, which are rich-in-H_2_S, into sulfur. SRU schematic process is shown in Figure 10. The content present in the tail gas is a crucial value to guarantee steady production and control airflow. Residual H_2_S and SO_2_ composes the tail gas, and their measurement is vital. However, using online analyzers to measure tail gas is not feasible due to its weak robustness and frequent maintenance. In such a case, soft-sensing techniques can estimate SO_2_, for example, as an alternative.

A total of 10,081 samples were collected from the explored SRU process, but only 10% of samples are pointed as labeled. As in the first study-case, the unsupervised stage uses the unlabeled samples as follows: 80% for training, 20% for validation. The labeled dataset is split into three parts: 420 samples for training, 85 samples for validation, and 504 samples for testing. SRU process variables are defined in Table 3. Furthermore, the feature engineering strategy creates features that can include previous features to handle process dynamics. Moreover, soft-sensor inputs are formed as $X=[u\left(t\right),\cdots ,u\left(t-d_{x}\right),y\left(t-1\right),\cdots ,y\left(t-d_{y}\right)]$, where $d_{x}$ and $d_{y}$ represents the maximal delayed interval. In this work, $d_{x}=d_{y}=6$.

The calculated MI threshold th is 0.032. SAE consists of three AEs with 41, 35, and 29 hidden units, respectively. After grid search, the activation function is ReLU, the learning rate set to 0.000750, and the batch size set to 10. A 512 cell-units LSTM structure couples to the tail of the SAE for fine-tuning after the unsupervised stage, intending to learn dynamic behavior. Early stop, L2 regularization, and cross-validation are applied to avoid overfitting.

**Figure 11:**
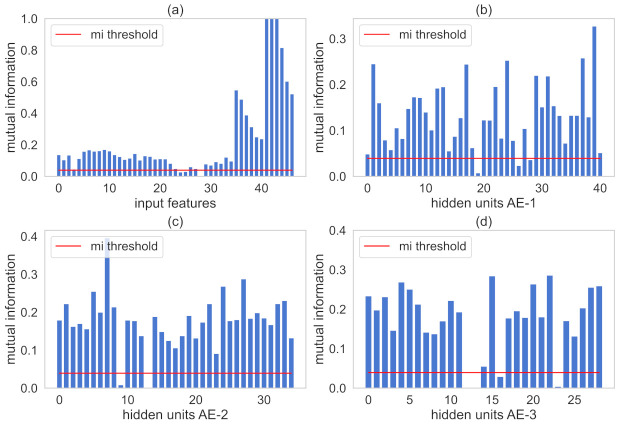
MI between input representations and the output variables for the SRU process. Subfigure (**a**) illustrates MI values of the input features. In addition, subfigures (**b**–**d**) indicate MI values hidden units present in AE-1, AE-2, AE-3, respectively.

Figure 11 plots MI values with th represented by the red line. MI numbers between all input variables and the target-output variable are illustrated in Figure 11a. Figure 11b–d illustrates the MI values among hidden learned representations of each AE and the output variable, respectively. The proposed method retains input variables and hidden information that showed MI numbers greater than th.

Table 4 shows the comparison details regarding the built soft sensors. As seen in a previous study case, PLS leads to the worst prediction since it does not handle non-linearities properly. As the unsupervised pre-training uses the unlabeled data, SAE-based models drive higher prediction performance than MLP and SVM. In contrast, MISAEL performs better than SAE-based models since only relevant representations are present and an LSTM structure enables dynamic features learning. Furthermore, a state-of-art SAE-based model named SIAE [30] was proposed and tested using the same SRU process used in this work. Regarding Table 4, MISAEL outperforms SIAE. As MISAEL, SIAE is an improved SAE-based model, but MISAEL takes advantage of dynamic LSTM-learning property, which establishes its enhanced performance. Additionally, eMISAEL and MISAEL demonstrate the lowest standard deviations (SD) of RMSE, which points to their stability under adversities. eMISAEL and MISAEL lead to the best performance results in comparison to the other traditional models MLP, SVR, PLS implemented in this work. Moreover, MISAEL outperforms the implemented SAE structure, and the state-of-art model SIAE [30] under same test conditions. The three major advantages that the MISAEL model employs: using unlabeled data for pre-training, MI-SAE with relevant representations for soft-sensing only, and LSTM for supervised fine-tuning. Furthermore, a k-fold cross-validation ensemble strategy is exploited by eMISAEL to boost MISAEL prediction performance.

**Figure 12:**
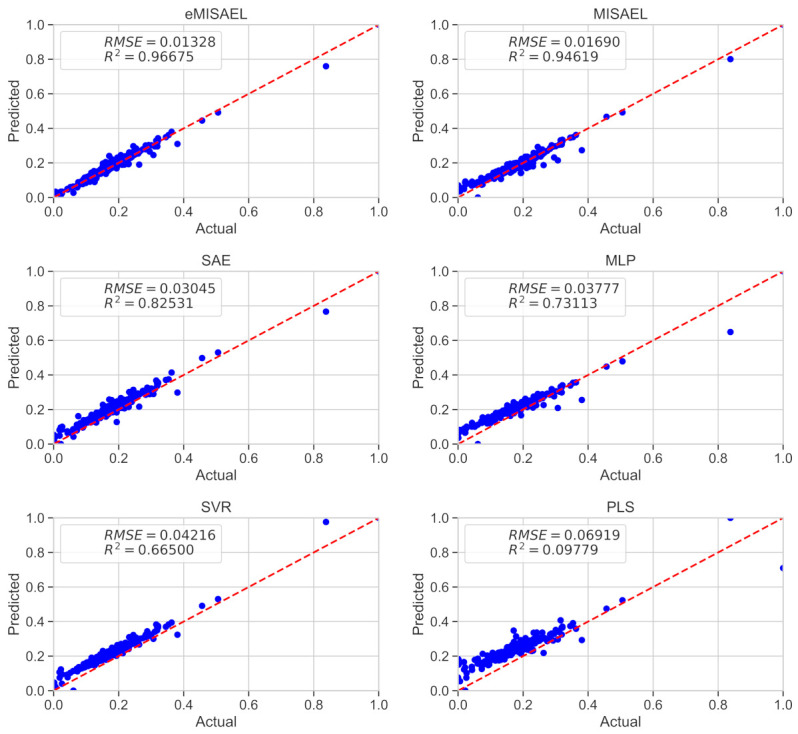
Real values of SO_2_ and the prediction using eMISAEL, MISAEL, SAE, MLP, SVR, and PLS soft sensors.

**Figure 13:**
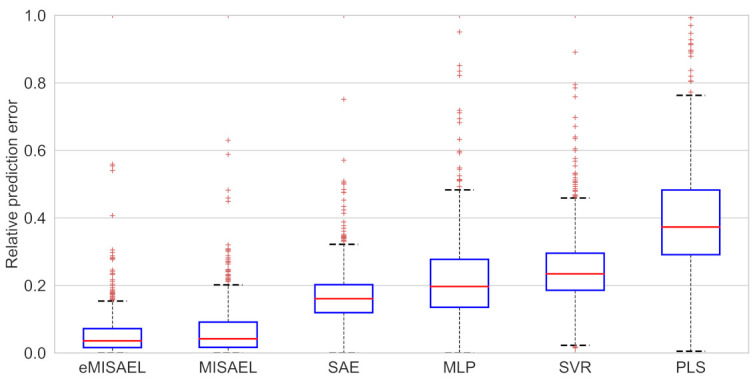
Relative prediction error of testing results for SRU process using eMISAEL, MISAEL, SAE, MLP, SVR, and PLS models, respectively.

The prediction results using the testing dataset are illustrated in Figure 12 by the use of parity plots. As predictable, eMISAEL and MISAEL show higher efficiency than the other studied soft sensors. The relative prediction errors are plotted in Figure 13 with six boxplots representing eMISAL, MISAEL, SAE, MLP, SVR, and PLS in ascending order, respectively. As in the first study case, the central red mark represents the median value, and the edges indicate the 25th and 75th percentiles. The “whiskers” represent maximum and minimum no outlier values. The wider the width of the box, the more dispersed prediction errors are. As the best models, eMISAEL and MISAEL are the models with the narrowest box ranges mainly because MISAEL extracts non-linear features from massive unlabeled data, retains only the relevant representations, and addresses process dynamicity. A few outliers are individually represented by red points in Figure 13, considering inherent industrial uncertainties [63]. High relative prediction errors result from inappropriate soft sensors due to both adverse initial parameters and outliers. Through, a satisfactory soft-sensor gives stationary relative prediction errors on the same data. Therefore, stationery predictions can reflect the robustness of the model [64]. MISAEL extracts non-linear features from massive unlabeled data, retains only the relevant representations for soft-sensor development, and addresses process dynamicity. Equal to the first study-case, MISAEL and eMISAEL implement a soft-sensor with improved performance, enhanced reliability, and stronger robustness.

## 5. Conclusions

A novel dynamic soft-sensing technique based on deep representative learning has been proposed and tested to predict industrial quality-related process variables. The proposed method MISAEL combines high-level feature extraction, relevant representations mining in the layers of the SAE, and dynamic features learning through LSTM models.

In the MISAEL model, a deep high-level feature extractor SAE exploited the massive amount of unlabeled data, which is not used for traditional methods, to improve soft-sensor representation capabilities in the unsupervised stage. To simulate a real scenario, 90% of the total data was considered unlabeled for training the SAE. Unsupervised SAE modeling does not guarantee relevant representations learning for soft-sensing purposes. A layer-by-layer MI-based approach analyzes the relationship between learned representations and the targeted-output values to highlight the most significant features. The procedure retains the relevant features and removes the irrelevant ones. Even with only highlighted information left, SAE may not address industrial process dynamicity. The LSTM model couples to the tail of the SAE in the supervised fine-tuning. A deep SAE-LSTM structure copes with the inherent dynamic behavior of the system.

Obtained results from two study cases prove that MISAEL presents improved prediction performance compared to traditional models, SAE-based soft sensors, and above-cited state-of-art data-driven models, which do not handle process dynamicity. According to the results, MISAEL is reliable, robust, and has improved performance compared to PLS, SVR, MLP, SAE and three state-of-art methods validated in the same case studies under same conditions. Furthermore, the proposed method has the proficiency to be applied for semi-supervised learning applications.

Despite the presented contributions, there are still improvements for future works. Targeted-output regularizers on the loss function would extract even better features, improving the proposed work. Another future intervention would be to apply techniques that highlight dynamic-related features on the unsupervised pre-training. Also, industrial study cases were used to implement the proposed method, but a soft-sensor proposal for a real industrial scenario may be a difficult task. It is worth mentioning that non-linearities, anomalies, and highly complex environments must be considered. However, the industrial study cases have been satisfactory and widely used to implement and evaluate models, and they are the base plants for very contributions in this field of research.

## Figures and Tables

**Table 1:** Description of debutanizer column process variables.

Variable 1	Variable Description	Unit
u1	Top temperature	${}^{\circ }$C
u2	Top pressure	kg/cm^2^
u3	Reflux flow	m^3^/h
u4	Flow to next process	m^3^/h
u5	Sixth tray temperature	${}^{\circ }$C
u6	Bottom temperature A	${}^{\circ }$C
u7	Bottom temperature B	${}^{\circ }$C
Output	Butane C4 content in IC5	-

**Table 2:** Prediction performance of debutanizer column soft-sensor models.

Model	RMSE±SD	$R^{2}$
PLS	0.0935 ± 0.00918	0.7594
SVR	0.0667 ± 0.00682	0.8777
MLP	0.0538 ± 0.00716	0.9204
SAE	0.0396 ± 0.00603	0.9568
HVW-SAE [33]	0.0308 ± NOT PROVIDED	0.9615
SSED [62]	0.0339 ± NOT PROVIDED	0.9557
MISAEL	0.0208 ± 0.00482	0.9880
eMISAEL	0.0194 ± 0.00331	0.9897

**Table 3:** Description of SRU process variables.

Variable 1	Variable Description	Unit
u1	Gas flow MEA GAS Air	Nm^3^/h
u2	Air flow AIR MEA	Nm^3^/h
u3	Secondary air flow AIR MEA 2	Nm^3^/h
u4	Gas flow in SWS zone Air	Nm^3^/h
u5	Air flow in SWS zone	Nm^3^/h
Output	Concentration of SO_2_ in the tail gas	-

**Table 4:** Prediction performance of SRU soft-sensor models.

Model	RMSE±SD	$R^{2}$
PLS	0.0692 ± 0.00874	0.0978
SVR	0.0422 ± 0.00598	0.6650
MLP	0.0378 ± 0.00612	0.7311
SAE	0.0305 ± 0.00561	0.8253
SIAE [30]	0.0279 ± NOT PROVIDED	0.7720
MISAEL	0.0169 ± 0.00499	0.9462
eMISAEL	0.0133 ± 0.00323	0.9668

## Data Availability

Original debutanizer column and SRU datasets are available http://www.springer.com/cda/content/document/cda_downloaddocument/9781846284793_material.zip?SGWID=0-0-45-349600-p168288081 (accessed on 8 January 2021).
